# Transit time flow measurement predicts graft patency in off-pump coronary artery bypass grafting upon 5-year angiographic follow-up

**DOI:** 10.1186/s13019-021-01716-3

**Published:** 2021-11-21

**Authors:** Caiwu Zeng, Xiaomi Li, Yan Dai, Ye Zhou, Chenglong Li, Nan Liu, Jiangang Wang

**Affiliations:** 1 Center for Cardiac Intensive Care, Beijing Anzhen Hospital, Capital Medical University, No.2 Anzhen Road, Chaoyang District, Beijing, 100029 People’s Republic of China; 2 Department of Cardiac Surgery, Beijing Anzhen Hospital, Capital Medical University, No.2 Anzhen Road, Chaoyang District, Beijing, 100029 Republic of China

**Keywords:** Off-pump coronary artery bypass grafting, Transit time flow measurement, Coronary angiography, Graft dysfunction

## Abstract

**Objective:**

This retrospective study sought to evaluate the efficacy of transit time flow measurement (TTFM) as a means of predicting bypass graft patency as assessed by coronary artery angiography upon 5-year follow-up.

**Methods:**

Of 311 patients undergone isolated off-pump coronary artery bypass graft surgery from January 2014 through December 2014, 202 (65%) underwent both intraoperative TTFM and angiography at follow-up. 610 grafts, 202 left internal mammary artery grafts and 408 saphenous vein grafts were checked. Any grafts that exhibited Fitzgibbon type B or O lesions upon angiographic evaluation were considered to be failing. Receiver operating characteristic curves were used to identify the optimal TTFM values for predicting graft patency.

**Results:**

A total of 610 grafts were included in this analysis, including 202 LIMA grafts and 408 SV grafts, of which 107, 129, 129, and 43 anastomosed to DIAG, OM, PDA, and PLA, respectively. LIMA, DIAG, OM, PDA, and PLA bypass grafts had overall patency rates of 95.0%, 74.8%, 73.6%, 71.5%, and 74.4%, respectively, upon 5-year follow up. No significant differences in TTFM values (MGF, PI, and DF) were observed when comparing outcomes associated with individual or sequential SV grafting. MGF was found to be predictive of graft failure regardless of the target vessel (*P* < 0.05). While PI was found to predict LIMA, OM, and PDA graft failure (*P* < 0.05), it was not associated with the failure of grafts associated with DIAG and PLA vessels. Similarly, DF was found to predict OM and PDA graft failure (*P* < 0.05), but was not significantly associated with the failure of grafts associated with LIMA, DIAG, or PLA vessels.

**Conclusion:**

LIMA bypass grafts were associated with better 5-year graft patency relative to SV bypass grafts. Similar graft patency rates were observed for both individual and sequential bypass grafts. MGF was able to predict bypass graft failure in patients that underwent off-pump CABG surgery.

## Background

Coronary artery bypass graft (CABG) surgery outcomes have significantly improved over the last 50 years [1], with this treatment remaining the optimal treatment for those with complex multivessel disease [2]. Intraoperative graft patency is a primary determinant of the short- and long-term success of CAGB surgery [3]. While coronary artery angiography (CAG) is the gold-standard approach used to assess graft patency, it can be an inconvenient and invasive procedure when conducted intraoperatively. As such, intraoperative graft function is most often assessed based upon transit time flow measurement (TTFM) values, which have the potential to significantly improve CABG procedure quality and patient clinical outcomes [6].

TTFM is typically used to evaluate intraoperative graft patency in accordance with guidelines published in 2010 [4], which additional support from the 2018 ESC/EACTS Guidelines on myocardial revascularization that provided a class-IIa recommendation for the use of TTFM for intraoperative graft assessment [5].

Few published studies to date, however, have evaluated the reliability of TTFM as a means of predicting long-term CAG graft patency findings in patients undergoing off-pump CABG surgery. This study was therefore designed to assess the ability of TTFM parameters to predict 5-year postoperative graft patency outcomes in off-pump CABG patients.

## Materials and method

### Patients and study design

Between January 2014 and December 2014, 311 total patients underwent isolated off-pump CABG surgery at Beijing Anzhen Hospital, Capital Medical University. Patients included in the present study were those with stable angina, a left ventricle ejection fraction (LVEF) ≥ 50%, and a left ventricular end-diastolic diameter (LVEDD) of ≤ 60 mm.

### Operative procedures

Median sternotomy, standard cannulation, and off-pump procedure stabilizers were employed for the treatment of all patients. Left internal mammary artery (LIMA) grafts were harvested as pedicles, while saphenous vein (SV) grafts were harvested via an open technique. End-to-side anastomoses were conducted in a continuous manner using 6–0 sutures for proximal aortic connections during partial aortic clamping and using 7–0 sutures for the terminal bypass. Side-to-side anti-parallel anastomoses were conducted in a continuous manner using 7–0 sutures for the sequential bypasses.

### Intraoperative graft flow measurement

A TTFM (VQ1001; Medi-stim AS, Oslo, Norway) approach was used to measure intraoperative graft flow parameters, including MGF, PI, and the DF. MGF was measured in mL/min, while PI was measured as the difference in peak systolic flow minus peak diastolic flow divided by the median flow and was used to estimate graft resistance. DF was measured as the percentage of the total flow during diastole. These TTFM procedures were recorded intraoperatively immediately prior to sternal closure with the transit-time flowmeter instrument and an appropriately sized probe that was able to fit tightly within the graft without causing compression. Measurements were made when the patient exhibited stable hemodynamics and a mean blood pressure of 70–90 mmHg.

### Postoperative follow-up

After surgery, aspirin (100 mg/d orally) and atorvastatin calcium tablets (10 mg/d orally) were administered to all patients with first 24 h and continued after their discharge, and clopidogrel (75 mg/d, orally) was added on the first postoperative day, and continued thereafter for 1 year. Other medications were prescribed as necessary. Postoperative follow-up was obtained via phone call or direct contact with patients or their families, and all patients underwent annual routine clinical assessment. At the 5-year follow-up time point, 28 patients had been lost to follow-up and 81 refused to undergo CAG. The remaining 202 patients (65%) consented to undergo CAG, and therefore were enrolled in our final study.

### Postoperative angiography

Two or more cardiologists independently analyzed angiographic recordings from each patient that had been collected using standard views, with grafts being assessed as per the Fitzgibbon classification system [7]. Each coronary anastomosis was considered to correspond to the distal end of a single bypass graft, regardless of trunk configuration. Excellent and unimpaired grafts were those with a grade A designation, whereas type B or type O grafts were considered to be occluded.

### Statistical analysis

Continuous data were given as means ± standard deviation and were compared via unpaired Student’s t-tests. Categorical variables were given as frequencies and percentages, and were compared via chi-squared tests or Fisher’ exact test. Optimal MGF, PI, and DF cut-off values for the prediction of 5-year graft failure were determined using receiver operating characteristic (ROC) curves as the point nearest point to the best point (specificity = 1, sensitivity = 1; upper left corner) of these ROC curves. *P* < 0.05 was the significance threshold in this study, and SPSS v25 was used for all statistical testing.

## Results

### Off-pump CABG patient characteristics

Patient baseline characteristics are compiled in Table 1.

**Table 1:** Preoperative characteristic

Characteristic	Value
*Demographics*	
Age	58.6 ± 7.3 (38, 76)
Male (%)	159 (78.7%)
BMI (kg/m^2^)	25.8 ± 3.1 (15.4, 35.5)
*Cardiovascular risk factors*	
Hypertension	119 (58.9%)
Diabetes mellitus	66 (32.7%)
Hyperlipidemia	55 (27.2%)
Current smoker	118 (58.4%)
*Comorbidities*	
Previous neurological events	12 (5.9%)
Renal disease	5 (2.5%)
COPD	15 (7.4%)
*Coronary lesion*	
Single vessel disease	7 (3.5%)
Two-vessel disease	43 (21.3%)
Three-vessel disease	152 (75.2%)
*Angina class*	
CCS I-II	190 (94.1%)
LVEDD (mm)	48.3 ± 4.8 (34, 60)
LVEF, %	61.8 ± 5.9 (50, 77)
Euro SCORE	1.8 ± 0.8 (0, 4)

BMI, body mass index; COPD, chronic obstructive pulmonary disease; CCS, Canadian Cardiovascular Society; LVEDD, left ventricular end diastolic diameter; LVEF, left ventricular ejection fraction

### Graft distributions

In total, 610 total grafts (3.01 grafts/patient) were included in the present study, including 202 LIMA grafts anastomosed to the LAD, and 408 SV grafts that were anastomosed to DIAG, OM, PDA, and PLA in 107, 129, 129, and 43 cases, respectively. A total of 66 patients were bypassed with a single distal SV graft, while 342 distal targets were sequentially bypassed with 141 SV grafts, for a combined total of 408 distal anastomoses with 207 SV graft conduits. Among patients that underwent sequential bypass, 85, 52, and 4 underwent double, triple, and quadruple sequential SV bypass grafting, respectively.

### TTFM parameters

No significant differences in TTFM parameters (MGF, PI, and DF) were observed when comparing individual and sequential SV grafting (Table 2), enabling us to analyze SV grafts in different distributions without taking grafting technique into consideration during these analyses.

**Table 2:** TTFM values of different bypass grafting technique in SVG

Variable	Grafting technique	MGF	PI	DF
DIAG	Individual grafting	23.9 ± 15.4 (8, 70)	1.8 ± 0.7 (1, 3)	71.5 ± 8.4 (55, 87)
	Sequential grafting	31.8 ± 21.0 (5, 89)	2.5 ± 0.9 (1.6)	71.6 ± 10.1 (41, 97)
OM	Individual grafting	28.8 ± 20.5 (6, 71)	2.8 ± 1.9 (1, 9)	66.1 ± 11.7 (47, 87)
	Sequential grafting	31.5 ± 21.4 (4, 125)	2.5 ± 1.2 (1, 11)	69.6 ± 9.9 (48, 92)
PDA	Individual grafting	26.5 ± 17.7 (9, 74)	2.7 ± 1.3 (1, 7)	62.1 ± 12.2 (27, 76)
	Sequential grafting	27.4 ± 21.1 (3, 86)	2.6 ± 1.7 (1, 10)	65.1 ± 9.7 (43, 85)
PLA	Individual grafting	26.3 ± 10.6 (10, 41)	2.2 ± 0.7 (1, 4)	70 ± 11.2 (58, 82)
	Sequential grafting	28.2 ± 15.8 (7, 73)	2.2 ± 1.2 (1, 6)	66.4 ± 12.5 (42, 98)

DIAG, diagonal artery; OM, obtuse marginal branch of circumflex artery; PDA, posterior descending artery; PLA, left posterior artery; MGF, mean graft flow; PI, pulsatile index; DF, diastolic filtration

TTFM values associated with different graft types are shown in Table 3. MGF and PI values were significantly higher and lower respectively in patent grafts relative to occluded grafts regardless of the target vessel (*P* < 0.05). While DF were significantly better in all patent OM and PDA grafts relative to occluded OM and PDA grafts (*P* < 0.05), no significant differences in these values were noted when comparing patent and occluded grafts associated with LIMA, DIAG, and PLA vessels.

**Table 3:** TTFM values of bypass graft

	MGF (mL/min)	PI	DF (%)
*LIMA*			
Patent (n = 192)	35.6 ± 21.4 (6, 130)	2.2 ± 0.8 (0.9, 5.0)	71.0 ± 8.5 (35, 99)
Occlusion (n = 10)	14.3 ± 5.8 (10, 27)	3.2 ± 1.4 (1.5, 5.8)	68.5 ± 9.1 (56, 80)
*P* value	< 0.001	0.049	0.357
*DIAG*			
Patent (n = 80)	36.6 ± 20.0 (14, 89)	2.3 ± 0.9 (0.9, 5.7)	71.8 ± 8.3 (51, 97)
Occlusion (n = 27)	12.8 ± 6.5 (5, 30)	2.7 ± 1.0 (1.2, 4.4)	70.8 ± 13.1 (41, 96)
*P* value	< 0.001	0.044	0.702
*OM*			
Patent (n = 95)	37.4 ± 21.1 (15, 125)	2.3 ± 1.0 (0.5, 8.6)	71.4 ± 9.3 (49, 92)
Occlusion (n = 34)	13.6 ± 6.3 (4, 34)	3.4 ± 1.7 (0.9, 10.6)	62.1 ± 9.9 (47, 83)
*P* value	< 0.001	0.001	< 0.001
*PDA*			
Patent (n = 91)	32.3 ± 19.9 (10, 86)	2.3 ± 1.3 (0.8, 9.6)	66.5 ± 9.8 (27, 85)
Occlusion (n = 38)	7.9 ± 2.8 (3, 13)	3.9 ± 2.1 (1.3, 10.0)	57.5 ± 9.0 (40, 73)
*P* value	< 0.001	< 0.001	0.005
*PLA*			
Patent (n = 32)	32.5 ± 14.2 (16, 73)	2.0 ± 1.0 (0.3, 4.8)	67.0 ± 9.9 (44, 83)
Occlusion (n = 11)	14.2 ± 5.6 (7, 25)	2.9 ± 1.4 (1.3, 6.3)	67.4 ± 16.5 (42, 98)
*P* value	< 0.001	0.021	0.925

LIMA, left internal mammary artery; SVG, saphenous vein graft; LAD, left anterior descending artery; DIAG, diagonal artery; OM, obtuse marginal branch of circumflex artery; PDA, posterior descending artery; PLA, left posterior artery; MGF, mean graft flow; PI, pulsatile index; DF, diastolic filtration

### Angiographic outcomes

**Fig. 1:**
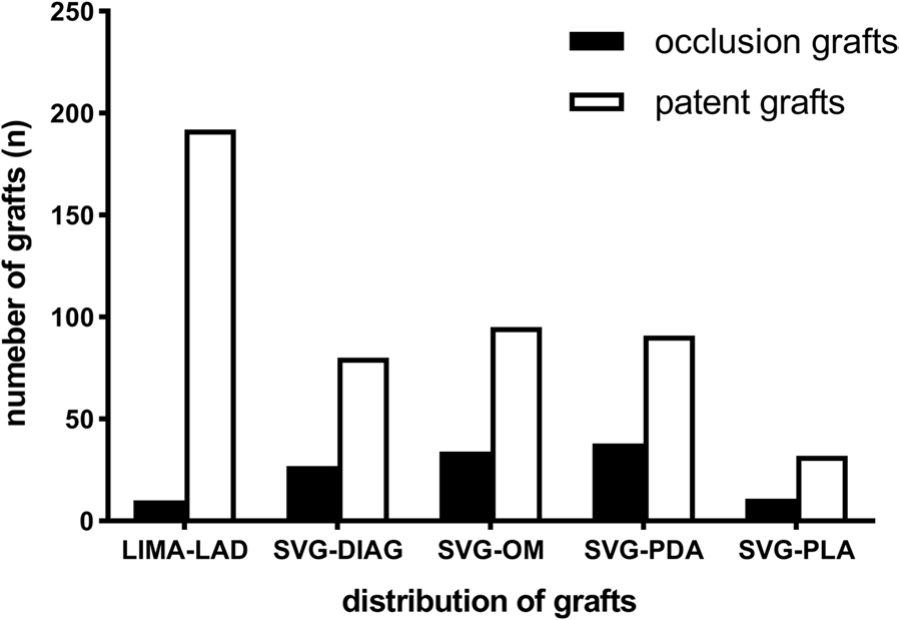
Distribution and angiographic outcomes of bypass grafts. LIMA, left internal mammary artery; SVG, saphenous vein graft; LAD, left anterior descending artery; DIAG, diagonal artery; OM, obtuse marginal branch of circumflex artery; PDA, posterior descending artery; PLA, left posterior artery

**Fig. 2:**
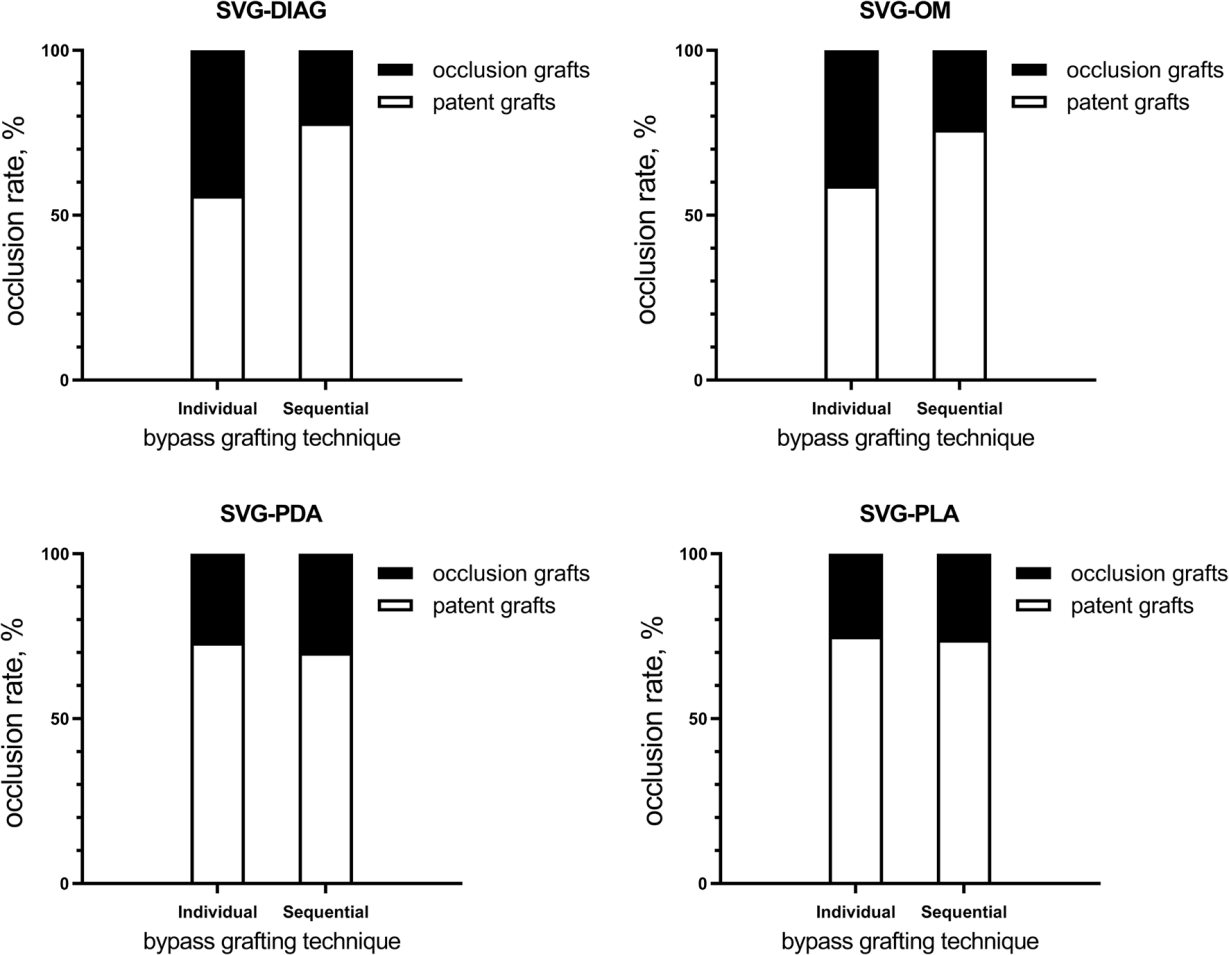
Angiographic outcomes of different bypass grafting technique in SVG. SVG, saphenous vein graft; DIAG, diagonal artery; OM, obtuse marginal branch of circumflex artery; PDA, posterior descending artery; PLA, left posterior artery

Postoperative 5-year graft patency rates for bypassed grafts in LAD, DIAG, OM, PDA, and PLA vessels were 95.0%, 74.8%, 73.6%, 71.5%, and 74.4%, respectively (Fig. 1). SV grafts were divided into subgroups based upon the employed bypass grafting approach (Fig. 2). No significant differences in SV graft patency were observed for DIAG, OM, PDA, or PLA grafts (*P* = 0.064, 0.137, 0.751, 1, respectively) when comparing individual and sequential grafting.

### ROC curve analyses

**Fig. 3:**
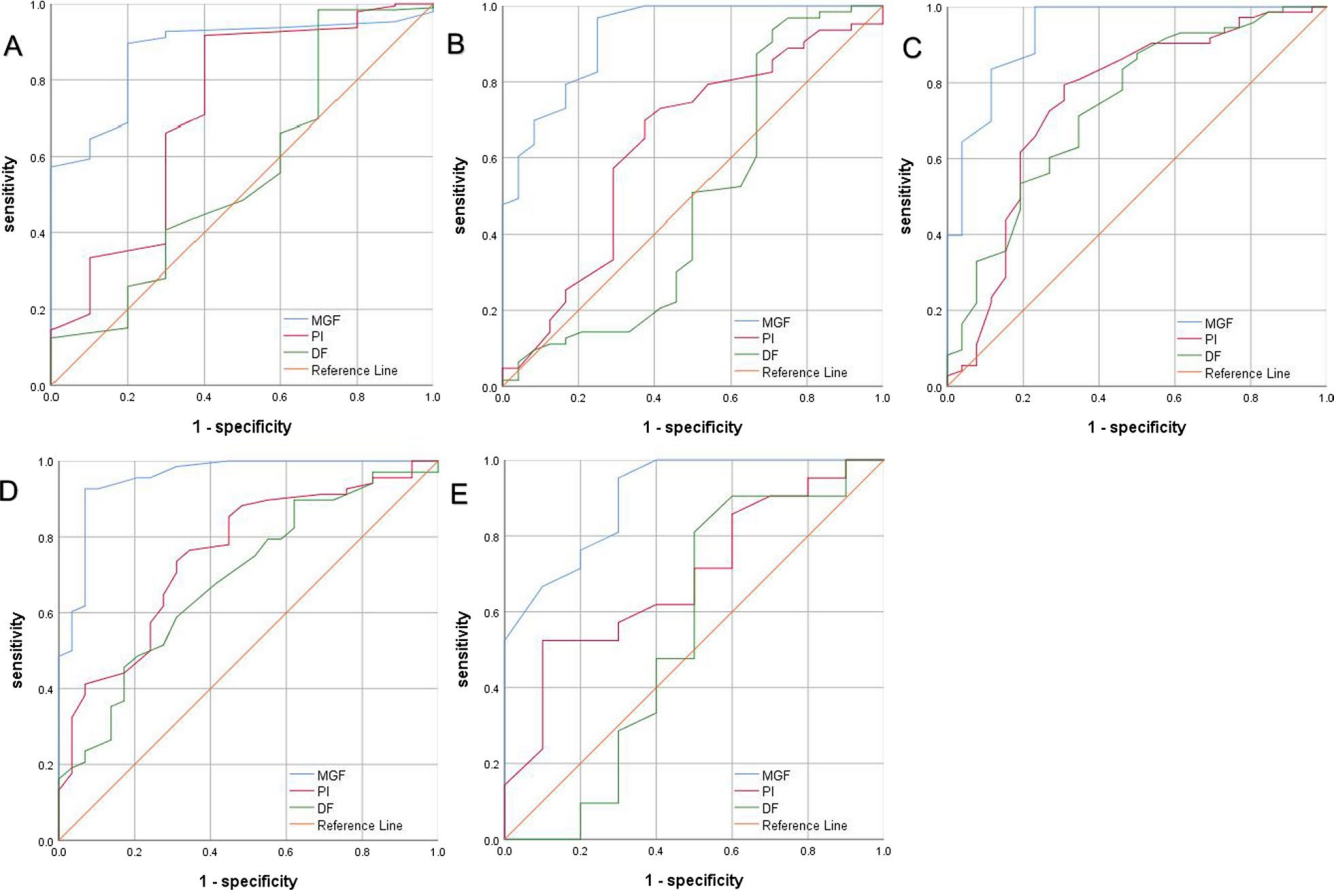
ROC curve of bypass graft. **A** ROC curve of LIMA; **B** ROC curve of DIAG; **C** ROC curve of OM; **D** ROC curve of PDA; **E** ROC curve of PLA. ROC, receiver operating characteristic; LIMA, left internal mammary artery; DIAG, diagonal artery; OM, obtuse marginal branch of circumflex artery; PDA, posterior descending artery; PLA, left posterior artery; SVG, saphenous vein graft; MGF, mean graft flow; PI, pulsatile index; DF, diastolic filtration

ROC curves corresponding to different graft types are shown in Fig. 3 and Table 4. ROC curve analyses of intraoperative TTFM values revealed that MGF (AUC: 0.875; *P* < 0.001) and PI (AUC: 0.725; *P* = 0.017) values were able to predict LIMA graft failure. The optimal MGF cut-off value for predicting 5-year graft failure was found to be 14.5 mL/min, with a sensitivity of 89.6% and a specificity of 80.0%. The optimal PI cut-off value for predicting 5-year graft failure was found to be 3.45 mL/min, with a sensitivity of 91.7% and a specificity of 60.0%.In contrast, DF (AUC: 0.560; *P* = 0.525) offered negligible predictive value for LIMA graft patency rates.

**Table 4:** ROC analysis of the TTFM

Variables	Cut-off	AUC	95% CI	*P*-value	Sensitivity	Specificity
*LIMA*						
MGF	14.5	0.875	0.789–0.961	< 0.001	0.896	0.800
PI	3.45	0.725	0.536–0.914	0.017	0.917	0.600
DF	57.5	0.560	0.357–0.762	0.525	0.984	0.300
*DIAG*						
MGF	14.5	0.921	0.857–0.985	< 0.001	0.968	0.750
PI	2.45	0.633	0.495–0.772	0.056	0.698	0.625
DF	60.5	0.484	0.325–0.642	0.816	0.937	0.292
*OM*						
MGF	14.5	0.935	0.876–0.993	< 0.001	1	0.769
PI	2.85	0.751	0.628–0.873	< 0.001	0.795	0.692
DF	60.5	0.737	0.621–0.853	< 0.001	0.877	0.500
*PDA*						
MGF	13.5	0.956	0.911–1	< 0.001	0.926	0.931
PI	2.65	0.755	0.651–0.858	< 0.001	0.735	0.690
DF	67.5	0.689	0.576–0.802	0.003	0.456	0.828
*PLA*						
MGF	16.5	0.905	0.794–1	< 0.001	0.952	0.700
PI	1.85	0.690	0.495–0.886	0.091	0.524	0.900
DF	60.5	0.545	0.287–0.803	0.688	0.810	0.500

The ROC analysis representing the cut-off graft flow for predicting 5-year Follow-up graft failure

ROC, receiver operating characteristic; LIMA, left internal mammary artery; SVG, saphenous vein graft; MGF, mean graft flow; PI, pulsatile index; DF, diastolic filtration; AUC, area under curve; CI, confidence interval

For SV grafts, MGF values were found to be predictive of graft failure regardless of the target vessel (*P* < 0.05). Optimal MGF cut-off values for DIAG, OM, PDA, and PLA grafts were 14.5 mL/min, 14.5 mL/min, 13.5 mL/min, and 16.5 mL/min, respectively, with respective sensitivity values of 96.8%, 100%, 92.6%, and 95.2%, and with respective specificity values of 75.0%, 76.9%, 93.1%, and 70.0%. While PI was found to offer value as a means of predicting OM and PDA graft failure (*P* < 0.001), it offered no such predictive value for DIAG and PLA grafts. Similarly, while DF values were able to predict OM and PDA graft failure (*P* < 0.05), they were unable to predict the failure of DIAG or PLA grafts.

## Discussion

Herein, we found that LIMA bypass grafts were associated with superior rates of 5-year patency as compared with SV bypass grafts, with similar 5-year patency rates for sequential and individual SV grafts. Our primary finding is that intraoperative MGF can strongly predict 5-year LIMA and SV graft patency, whereas the PI and DF TTFM parameters cannot reliably predict these outcomes. The development of new distal coronary disease over 5 years such as neointimal hyperplasia and atherosclerotic degeneration, may be a factor making the PI not as useful in predicting late graft failure.

Relatively few studies to date use DF as a parameter to predict graft patency; although no significant differences were noted in the LIMA, DIAG, PLA vessels, the fact that there were significant DF values in OM and PDA grafts. This patency difference may reflect predominantly with left vs right sided grafts. The hemodynamic characteristics of a patent graft are resemblance to coronary circulation, and the flow curve pattern predominant is diastolic with less systolic peaks during the isovolumetric ventricular contraction. Because of the thinner walled right ventricular, the right coronary arteries are less compressed by ventricular than the left coronary arteries. Therefore, the right coronary territories has more blood flow during systole than the left coronary territories.

Previously published guidelines recommend intraoperative graft assessment [4, 5]. Specifically, MGF is recommended to remain > 20 mL/min, with a PI value of < 5. However, these guidelines fail to take graft type of anastomosis methodology into account, and they do not provide concrete cut-off values that can be used to predict graft failure. These recommendations were based on a retrospective analysis of 3-year follow-up data for 990 arterial grafts in patients that underwent on-pump CABG surgery [8]. As this study did not analyze SV grafts, these guidelines may not be well-suited to such grafts, and in the absence of any angiographic follow-up it is difficult to evaluate the relationship between intraoperative TTFM parameters and long-term graft patency. Furthermore, controversy remains regarding rates of graft patency associated with off-pump and on-pump CABG surgery. Hattler et al. [9] found on-pump CABG to be associated with excellent graft patency rates, whereas Puskas et al. [10] detected comparable patency rates when comparing on-pump and off-pump CABG outcomes.

The difference between off and on-pump flows. Typically, off-pump flows are less than on-pump because of the ischemia generated by the cross clamp of on-pump causing reactive hyperemia secondary to ischemia. The cut-off flows found in this study of ~ 13–16 mL/min are not much different from those suggested in the guidelines (> 20 mL/min) and may simply reflect the non-ischemic technique of off-pump coronary surgery.

Relatively few studies to date have compared TTFM measurements with angiographic follow-up data, and results from such analyses have been inconsistent. Singh et al. [11] for example, studied 156 patients that underwent CABG surgery and that were randomized to intraoperative graft assessment or no assessment groups, detecting no significant differences in graft patency rates as a function of whether or not intraoperative graft assessment was conducted. In contrast, Quin et al. [12] assessed outcomes for 2203 patients that underwent CABG surgery and found that FitzGibbon grade A patency was less frequently detected for grafts with low intraoperative MGF values relative to grafts with normal intraoperative flow. They also found FitzGibbon grade A patency to be negatively correlated with PI values. We also found MGF values to strongly predict the long-term patency of LIMA and SV grafts, whereas PI was only a reliable predictor of LIMA graft patency. Relatively few studies have examined relationships between graft patency rates and TTFM parameters in patients undergoing off-pump CABG surgery. One reason for this is the fact that many factors can influence the readouts from this analysis, including graft quality and type, the nature of the coronary artery, hemoglobin levels, and individual patient hemodynamics [13, 14].

All LIMA grafts in patients in the present study were anastomosed to the LAD, with an overall 5-year patency rate of 95.0%, while DIAG, OM, PDA, and PLA SV grafts exhibited 5-year patency rates of 74.8%, 73.6%, 71.5%, and 74.4%, respectively. LIMA grafts were associated with better 5-year patency rates relative to SV grafts in the present study. As long-term graft patency is maximized by the use of arterial grafts, and specifically the LIMA [15], all patients should receive a minimum of one arteria graft to the LAD barring exceptional circumstances [16]. SV graft patency rates for non-LAD targets, in contrast, are generally found to be suboptimal [17].

We observed similar patency rates when comparing individual and sequential SV bypass grafts. At present, there is no consensus regarding which of these grafting techniques is optimal. For example, Kim et al. conducted a study of 309 patients that underwent either sequential or individual SV CABG surgery, and concluded that sequential bypass grafting was associated with higher mean flow rates and with better mid-term patency relative to individual grafting [18]. This is in contrast to our findings and may be explained by the relatively short 14.8 month mean follow-up duration in this prior study. Over time, proximal anastomosis may be associated with higher rates of occlusion, potentially resulting in simultaneous myocardial ischemic events in multiple regions.

Herein we calculated optimal TTFM cut-off values for assessing graft failure risk in patients undergoing off-pump CABG surgery. For LIMA grafts, an MGF cut-off value of 14.5 mL/min was found to be able to strongly predict graft patency (AUC = 0.875).at the same time, a PI cut-off value of 3.45 offered a moderate ability for LIMA grafts (AUC = 0.725). For DIAG, OM, PDA, and PLA grafts, we calculated optimal MGF cut-off values of 14.5 mL/min (AUC = 0.921), 14.5 mL/min (AUC = 0.935), 13.5 mL/min (AUC = 0.956), and 16.5 mL/min (AUC = 0.905), respectively. PI cut-off values for SV grafts were inconsistent. However, there are so many factors affecting flow in a bypass graft. Such as: size, length, quality of conduit and of native artery, run-off quality of the coronary bed, mean arterial pressure, heart rate, competitive flow, viscosity of the blood. As we described in the article, the specificity of cut-off MGF value of LIMA, DIAG, OM, PDA, PLA was 80%, 75%, 77%, 93% and 70%, respectively. The threshold of diagnostic accuracy was relatively low, so the cut-off MGF value is insufficient to guide clinical practice. Our study suggests that grafts are obviously not patent by TTFM should be revised.

### Limitations

There are certain limitations to this study. For one, it was retrospective in nature rather than a randomized controlled trial. In addition, the sample size in the present study was small, and all off-pump CABG surgeries described herein were conducted at a single center. In addition, the measurements taken during surgery may not truly reflect the capacity of a given graft to carry flow, as these measurements can be influenced by many factors including cardiac recovery and graft spasm. Most of all, many factors affecting graft patency over time is this length of time passed before angiographic assessment.

## Conclusion

MGF was able to predict rates of 5-year LIMA and SV graft failure in patients undergoing off-pump CABG surgery. Our study suggests that grafts are obviously not patent by TTFM should be revised.

## Data Availability

The data used to support the findings of this study are available from the corresponding author upon request.

## Abbreviations

CABG: Coronary artery bypass grafting
TTFM: Transit time flow measurement
MGF: Mean graft flow
PI: Pulsatile index
DF: Diastolic filtration
LIMA: Left internal mammary artery
SVG: Saphenous vein graft
LAD: Left anterior descending artery
DIAG: Diagonal artery
OM: Obtuse marginal branch of circumflex artery
PDA: Posterior descending artery
